# Is delirium after stroke associated with dysregulation of hypothalamic pituitary axis?

**DOI:** 10.3389/fstro.2025.1584571

**Published:** 2025-06-26

**Authors:** Amanda J. Barugh, Alasdair M. J. MacLullich, Susan S. Shenkin, Michael Allerhand, Gillian E. Mead

**Affiliations:** ^1^Geriatric and Stroke Medicine, Royal Infirmary, Edinburgh, United Kingdom; ^2^Geriatric Medicine, Ageing and Health, Royal Infirmary, The University of Edinburgh, Edinburgh, United Kingdom; ^3^Health Care for Older People, Ageing and Health, Royal Infirmary, The University of Edinburgh, Edinburgh, United Kingdom; ^4^Statistical Consulting Unit, School of Mathematics, The University of Edinburgh, Edinburgh, United Kingdom; ^5^Stroke and Elderly Care Medicine, Ageing and Health, Royal Infirmary, The University of Edinburgh, Edinburgh, United Kingdom

**Keywords:** stroke, delirium, cognition, cortisol, hypothalamic-pituitary-adrenal (HPA)

## Abstract

Delirium after stroke is a serious condition associated with worse long-term cognition. However, the mechanism of delirium is not well understood. The aberrant stress response has been postulated as a mechanism for delirium.

**Aim:**

to explore the relationship between cortisol dysregulation and delirium over the first year after stroke in a prospective cohort study of patients admitted to an acute stroke unit.

**Methods:**

consecutive patients admitted to an acute stroke unit over a 1 year period were identified and recruited if they were aged age ≥ 60 years and not taking steroids. Patients with incapacity were included if proxy consent could be obtained. Baseline data included stroke severity, cognition, illness severity, and pre-stroke cognition. Patients were assessed at 1, 3, 5, 7, 14, 21, 28 days, 4 months and 12 months for delirium. Salivary samples were taken morning and evening for cortisol analysis.

**Results:**

of the 831 patients screened, 304 met inclusion criteria and of these 95 agreed to participant. Twenty-six (27%) had delirium at some point during the 12 months follow-up. Delirium was associated with increasing age (mean age 83.5 years vs. 74 years, *p* < 0.001), being female (62% vs. 23%, *p* = 0.013), not independent in pre-stroke activities of daily living (35% vs. 33%), higher IQCODE score median 3.56 vs. 3.19), worse stroke severity (median National Institute of Stroke Scale 5 vs. 8.5) *p* = 0.009) and having had a total anterior circulation stroke (*p* < 0.001). Univariable analyses identified several associations between delirium and cortisol in the first 28 days but not at 4 or 12 months. However, on multivariable analyses there were no significant associations between delirium and cortisol at any time point, e.g., odds ratio for median 9 am cortisol 0.95 (95% CI 0.89 to 1.01, *p* = 0.08).

**Conclusion:**

there was no independent association between delirium and cortisol dysregulation after stroke. If an association does exist, it is likely to be small.

## Background

Delirium is common after stroke; a 2019 systematic review reported a frequency of between 1.4 and 75.6% of patients (Stokholm et al., 2019). A subsequent 2019 study of over 700 patients reported that about a quarter developed delirium in an acute setting (Shaw et al., 2019). Delirium is independently associated with poorer functional outcomes (Rollo et al., 2022) and needing 24 h care (Pasińska et al., 2019). Delirium in the older population predicts later dementia (Richardson et al., 2021).

The underlying mechanisms of post-stroke delirium are uncertain. Hormonal abnormalities, including dysregulation of the hypothalamic-pituitary-adrenal (HPA) axis, have long been considered to be potentially important in its etiology (Cao et al., 2023; MacLullich et al., 2008). Treating patients in the intensive care unit setting with steroids is associated with longer term cognitive impairment (Cao et al., 2023). If HPA axis dysregulation (e.g., impaired negative feedback regulation of cortisol, loss of the circadian rhythm) is associated with delirium after stroke, and if this relationship is causal, then this could provide a target for the prevention and/or treatment of post-stroke delirium; and this might reduce the longer term risk of cognitive impairment and dementia after stroke.

Stroke is associated with a stress response and raised cortisol (Barugh et al., 2014). A 2024 systematic review identified 18 papers reporting associations between cortisol and cognitive and emotional consequences of stroke and most of these reported that patients with high cortisol levels on admission (acute phase of stroke) were more likely to experience cognitive decline and depression later in life (Wang et al., 2024). However, it is not known whether delirium mediates the relationship between raised cortisol and worse long term cognitive impairment. There are a few studies exploring the relationship between cortisol and delirium after stroke; but these were small, not all potential confounders were corrected for, and some measured cortisol only at a single time point after stroke (Gustafson et al., 1993; Fassbender et al., 1994; Marklund et al., 2004; Olsson, 1990).

Therefore, the aim of this study is to test the hypothesis that delirium after stroke is associated with higher circulating levels of cortisol, not just at a single time point after stroke, but at multiple time points in the first year after stroke.

## Methods

This was a prospective cohort study of patients admitted to an acute stroke unit in a teaching hospital from October 2012 for 1 year and followed up for a year. Inclusion criteria were clinically confirmed stroke, age ≥ 60 years and onset within previous 120 h (or time since last seen well for those occurring overnight) at the time of consent. Exclusion criteria were transient ischaemic attack, subarachnoid hemorrhage, current or recent use (within 6 months) of oral or inhaled corticosteroids, active alcohol withdrawal and inability to speak English prior to stroke. A clinical research fellow (AJB) approached potentially eligible patients; those with capacity consented for themselves, proxy consent was obtained from those without capacity. Easy access information materials were available for people with aphasia, thus maximizing the possibility that patients could consent for themselves, rather than requiring a proxy to consent on their behalf. Ethics approval was obtained from Scotland A Research Ethics Committee.

### Baseline clinical data

AJB collected the following data at time of recruitment: pathological stroke type (ischaemic or haemorrhagic), Oxfordshire Community Stroke Project classification (OCSP) and National Institute of Health Stroke Scale score (NIHSS). The OCSP was chosen as this is widely used in clinical practice in the UK and elsewhere in the world, as it provides a guide to potential causes of stroke and is useful in predicting prognosis. The NIHSS was chosen as this is used internationally to assess stroke severity. The National Adult Reading Test (NART) (McGurn et al., 2004) and The Informant Questionnaire on Cognitive Decline in the Elderly (IQCODE) (Jorm, 1995) were used to assess premorbid cognition. Heart rate, blood pressure, oxygen saturations and temperature, medications and admission blood results were recorded at the time of recruitment. The APACHE II (The Acute Physiology and Chronic Health Evaluation System II (Chikakuta, 1990) was used as a measure of current illness burden. Past medical history was extracted from case notes.

### Delirium assessments

Participants were assessed for delirium after the onset of stroke at 1, 3, 5, 7, 14, 21, 28 days, 4 months and 12 months. Those recruited after day 1 were seen from day 3 or day 5 onwards. Those discharged from hospital after a short admission were seen as per protocol whilst an in-patient and were then seen at home on day 28 and at 4 months and 12 months. AJB had been trained in screening and diagnosing delirium by two experienced raters.

The Confusion Assessment Method ICU (Ely et al., 2001) which includes the Richmond Agitation and Sedation Scale (RASS) (Sessler et al., 2002), and the Delirium Rating Scale-Revised-98 (Trzepacz et al., 2001) were used to identify delirium and its severity. The OSLA was used to assess alertness (Tieges et al., 2013). The Chart Confusion Assessment Method (Inouye et al., 2005) was also performed; this includes a question about a recent change or fluctuation in mental state, thus covering the non-assessed days. Attention, a core feature of delirium, was assessed by digit span (forwards and backwards). A validated computerized test for attentional deficits in delirium (Brown et al., 2011) was used for all participants except for those with a visual field deficit. A diagnosis of delirium was made based on the four DSM-IV criteria (acute onset or fluctuating course, inattention, disorganized thinking and altered level of consciousness).

### Cognition

At baseline, 1 month, 4 months and 12 months, cognition was assessed using the Montreal Cognitive Assessment MoCA (Nasreddine et al., 2005; Pendlebury et al., 2010).

At 1 month, 4 month and 12 month follow-up, a detailed neuropsychological battery was also administered; these data will be reported separately.

### Other outcome measures

At each of the follow-up visits (1 month, 4 months and 12 months), the Nottingham Extended Activities of Daily Activity score was performed (Lincoln and Gladman, 1992). We also extracted information from clinically performed brain imaging on lesion location, atrophy and white matter disease which has been reported separately. Thrombectomy was not performed on any of the patients as it had not been introduced into clinical practice at the time of the study. Those patients eligible for intravenous thrombolysis received it as part of standard clinical care.

### Salivary cortisol sampling

Saliva was collected by the research fellow at recruitment at 0930 and 1530, and at the same times on each subsequent assessment day. For follow-ups (1 month, 4 months and 12 months) participants with intact swallowing who could follow written instructions at hospital discharge were sent saliva swab and instructions for collection on the day of the researcher's visit. They collected a saliva sample on waking on the morning of the researcher's visit, stored it in the fridge, and the research fellow collected the second sample at around 1530. For those who were unable to collect their own saliva sample, two visits were made to the participant's home, one in the morning and one in the afternoon. Samples were collected using Salimetrics Oral Swabs (for those with an intact swallowing mechanism, as judged by the clinical team looking after the participant) or Salimetrics Children's Swabs (for those with dysphagia), placed in a sealed tube, centrifuged for 10 min at 3,000 rpm, at room temperature, and then stored at −80°C. The Dresden LabService Gmbh (Tatzberg 47-49, 01307, Dresden, Germany) performed the analysis using Enzyme-Linked Immunosorbent Assays (ELISA). Although the 12 month measurement was unlikely to be a causal factor in earlier delirium (due to the temporal nature of any relationship) we decided to include it so that we could use the data for novel hypothesis generating ideas about the relationship between cortisol dysregulation a year after stroke and cognitive impairment.

### Statistical analysis

Analyses were performed using Statistical Package for the Social Sciences (SPSS) version 14 and 19, except for the random effects modeling using the R statistics package.

### Power calculation

Assuming a sample size of 120 participants, and a delirium incidence of 30–40%, the main analyses were comparisons of cortisol levels at each time point between patients with (~*N* = 40) and without (~*N* = 80) delirium. With alpha set at *p* = 0.05, we had >80% power to detect medium-sized (Cohen's *d* = 0.5) differences.

### Statistical analysis

Participants were divided into two groups, those who developed delirium at *any time point* and those who did not, and descriptive analyses were performed for the two groups.

Morning (am) and afternoon (pm) cortisol levels from each time point were plotted on simple line graphs. Am:pm ratios were calculated and plotted to explore cortisol variability throughout the day. Histograms to demonstrate the distribution of the data were plotted. A Mann Whitney *U* test was performed to compare unadjusted cortisol levels between the two groups.

### Bivariable analysis

Spearman's correlations were used for the relationship between cortisol levels and baseline characteristics including age, NIHSS score, admission APACHE II score, IQCODE and Charlson Comorbidities Index. Biserial correlations were used for cortisol levels and sex (binary outcome, male or female). Spearman's correlations were used for the relationship between cortisol levels and continuous measures of delirium taken on the same day as the cortisol samples (the DRS-R98 and the Del-box); and biserial correlations were used for the relationship between cortisol and dichotomised (positive or negative) measures of delirium (CAM-ICU (taken on the same day as the cortisol samples) and delirium diagnosis at any time point during the 12 months).

For those who developed delirium, Spearman's correlations were used to explore the relationship between cortisol levels on the day of delirium diagnosis and delirium severity (as measured by the DRS-R98) on the same day. Finally, biserial correlations were used to investigate the relationship between the peak morning and peak afternoon cortisol levels during the first 14 days after stroke (morning and afternoon samples taken on the same day) and diagnosis of delirium at any point throughout the study period and to explore the relationship between median cortisol in the first 14 days after stroke and delirium diagnosis.

### Multivariable analysis

Multicollinearity was tested using variance inflation factor. Binary logistic regression, using the enter method, was used to explore the relationship between delirium diagnosis at any time throughout the study (as a binary outcome, yes or no) and median salivary cortisol levels in the week after stroke (day 0–7). Models were constructed using median morning and median afternoon cortisol levels. The first week was chosen as cortisol levels were found to be high in the first week after stroke in a systematic review (Barugh et al., 2014). A second set of models, using binary logistic regression were constructed using the peak cortisol levels (morning, afternoon and the ratio of morning to afternoon) during the first 14 days after stroke. The first 14 days was used for this analysis (rather than the first 7 days) in order to capture any later peaks. Important covariates selected a priori were age, sex, NIHSS score, IQCODE, Charlson Comorbidities index and APACHE II score, thus the analysis was designed to estimate the effect of cortisol on delirium independent of stroke severity, acute illness severity, chronic illness burden and prior cognitive impairment.

Finally a generalized linear random effects model was fitted to investigate the effect of cortisol (morning, afternoon and ratio of morning: afternoon) over time on presence or absence of delirium. The outcome variable was presence of delirium at any point during the study (yes or no), and the predictor variable was salivary cortisol level. The model was specified with binomial errors and a logit link function, which is appropriate for predicting the odds of group membership using a binary outcome variable (in this instance delirium, yes or no). The analysis was conducted using the GLMER function in the LME4 R statistics package.

There were multiple tests performed and so we expected some false positives. Thus, in our results tables, we have provided *p* values, and indicated significance, at the *p* < 0.05 level, at the *p* < 0.01 level and the *p* < 0.001 level.

## Results

**Figure 1:**
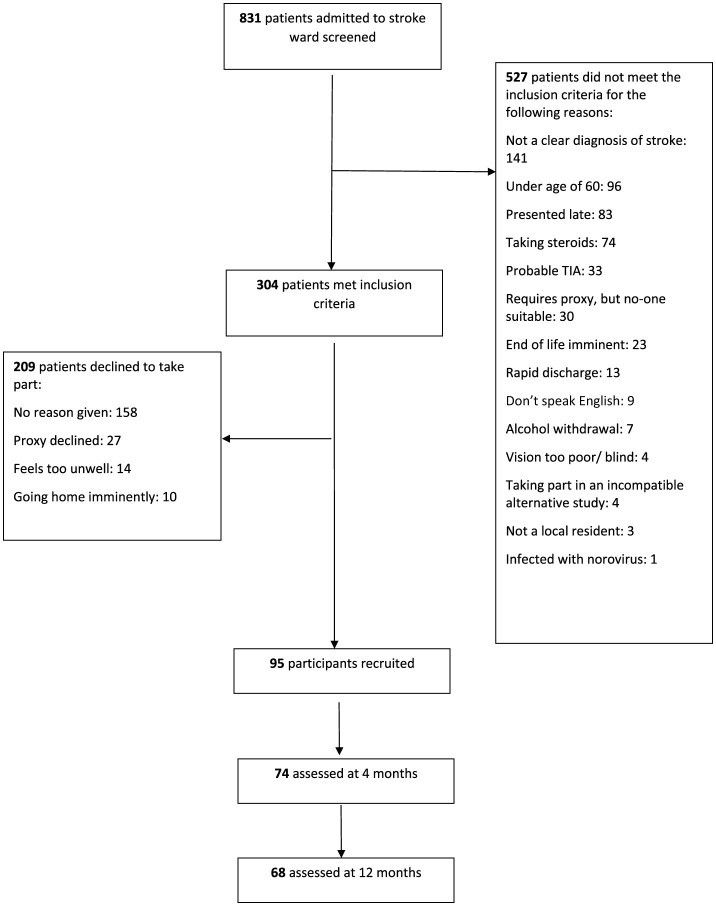
Flow diagram for recruitment.

Of the 831 patients screened during the recruitment period, 304 met inclusion criteria and of these 95 agreed to participate (Figure 1). An acute stroke lesion was visible in 40 (42%) participants (32 had ischaemic lesions and 8 had primary intracerebral hemorrhage on brain computed tomography [ICH]). All patients received stroke unit care, none received thrombectomy as this had not been introduced into our service at the time of recruitment, and those with ischaemic stroke presenting within 4.5 h were considered for thrombolysis. Seventy-four participants were followed up at 4 months and 68 were followed up at 12 months (attrition was mainly due to death, ongoing ill health and disability). Twenty-six (27%) had delirium at some point throughout the 12 month study period. Delirium was diagnosed at a median of day 5 after stroke (IQR day 4-day 7); median delirium severity score (DRS-R98) was 22 (IQR 16-26). Delirium was associated with increasing age, being female, less independent in pre-stroke ADLs, pre-existing cognitive impairment (IQCODE score), increasing stroke severity and having had a TACS on univariable analysis (Table 1).

**Table 1:** Characteristics of those developing delirium at at least one time point during the study.

**Characteristic**	**Whole cohort (*n* = 95) Median (interquartile range)**	**Delirium (*n* = 26) Median (interquartile range)**	**No delirium (*n* = 69) Median (interquartile range)**	***p***
Age	77 years (71–84)	83.5years (79–85.3)	74 years (68.5–82)	<0.001^***^^a^
Male sex	*N* = 56 (59%)	*N* = 10 (38%)	*N* = 46 (67%)	0.013^**^^b^
Time in formal education	11 years (11–13)	11 years (10–12)	11 years (11–14)	0.036^*^^a^
Independent in ADLs pre-stroke	*N* = 81 (85%)	*N* = 17 (65%)	*N* = 64 (67%)	0.006^**^^b^
IQCODE score	3.25 (3–3.69)	3.56 (3.19–4.6)	3.19 (3.0–3.7)	0.022^*^^a^
NART score	35(29.25–41)	34.5 (25.25–40.25)	36 (29.75–41.25)	0.356^a^
NIHSS score	5 (4–8)	8.5 (5–12.75)	5 (3–7)	0.009^**^^a^
APACHE II score	8 (6–10)	9 (7.75–12.25)	7 (6–10)	0.350^a^
Charlson co-morbidity index	2 (1–4)	2 (1.75–4)	2 (1–4)	0.572^a^
Stroke type (OCSP)	TACS 15 PACS 35 LACS 33 POCS 12	TACS 12 PACS 8 LACS 5 POCS 1	TACS 3 PACS 27 LACS 28 POCS 11	<0.001^***^^b^

a Mann-Whitney U test.

b Pearson chi-square.

* Significant at the 0.05 level.

** Significant at the 0.01 level.

*** Significant at the < 0.001 level.

ADLs, activities of daily living; APACHE II, acute physiology and chronic health evaluation II; NART, National adult reading test; NIHSS, National Institute of Health Stroke Score; OCSP, Oxfordshire community stroke project classification; TACS, total anterior circulation syndrome; PACS, partial anterior circulation syndrome; POCS, posterior circulation syndrome; LACS, lacunar syndrome.

**Figure 2:**
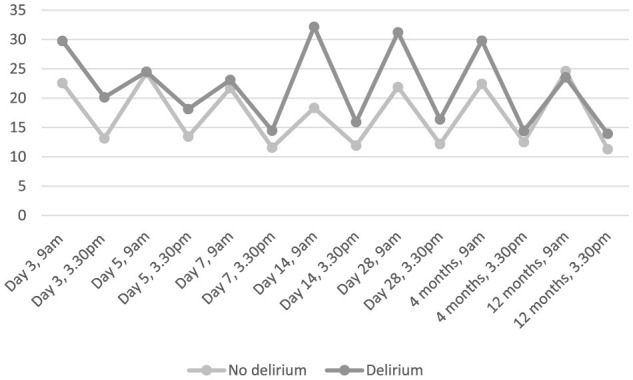
Median salivary cortisol, nmol/L, over time, in those who developed delirium at any time compared with those who did not.

The number of patients available for cortisol measurements ranged from 37 (9 am cortisol day 14) to 77 (9 am cortisol day 28). As expected, median cortisol levels were higher in the morning or afternoon at each time point and generally higher in those with delirium (Table 2 and Figure 2). Of the 21 cortisol measurements shown, there were statistically significant associations (*p* < 0.05) between delirium and seven of these measurements.

**Table 2:** Cortisol levels at each time point compared with those who developed delirium at any time point compared with those who didn't.

**Cortisol sample (nmol/L)**	**No delirium (*****n*** = **69)**			**Delirium (*****n*** = **26)**			***p* ^♢^**
	***n***	**Median**	**IQR**	***n***	**Median**	**IQR**	
9 am cortisol day 3	39	22.58	14.16–30.41	9	29.76	25.10–38.89	0.04^*^
3.30 pm cortisol day 3	37	13.15	8.53–18.08	8	20.14	10.83–54.81	0.05
Ratio am:pm cortisol day 3	36	1.72	1.26–2.28	7	1.41	1.02–3.03	0.55
9 am cortisol day 5	49	24.33	18.5–30.42	21	24.53	18.81–33.20	0.74
3.30 pm cortisol day 5	48	13.45	9.3–17.57	18	18.18	13.11–20.95	0.04^*^
Ratio am:pm cortisol day 5	46	1.87	1.28–2.58	18	1.44	0.95–1.75	0.02^*^
9 am cortisol day 7	35	21.72	16.75–33.06	17	23.12	16.07–27.66	0.66
3.30 pm cortisol day 7	32	11.58	9.25–16.73	15	14.48	10.95–16.96	0.58
Ratio am:pm cortisol day 7	31	1.51	1.16–2.46	14	1.57	1.40–2.26	0.93
9 am cortisol day 14	26	18.35	15.17–24.51	11	32.18	17.15–43.17	0.01^**^
3.30 pm cortisol day 14	19	11.92	9.32–15.86	11	15.94	12.80–20.61	0.02^*^
Ratio am:pm cortisol day 14	18	1.65	1.24–2.38	9	1.77	1.02–2.21	0.94
9 am cortisol day 28	59	21.92	17.88–30.82	18	31.25	16.48–39.06	0.22
3.30 pm cortisol day 28	60	12.19	8.95–15.66	14	16.41	11.07–22.54	0.03^*^
Ratio am:pm cortisol day 28	54	1.93	1.4–2.93	11	1.66	1.41–2.00	0.48
9 am cortisol 4 months	50	22.43	15.65–27.03	15	29.81	18.66–34.15	0.04^*^
3.30 pm cortisol 4 months	60	12.52	7.9–15.72	14	14.46	8.93–19.11	0.28
Ratio am:pm cortisol 4 months	48	1.83	1.16–2.66	11	1.98	1.41–2.21	0.85
9 am cortisol 12 months	53	24.61	16.64–37.6	15	23.57	18.2–36.5	0.91
3.30 pm cortisol 12 months	53	11.33	6.22–22.17	14	13.94	7.94–22.56	0.42
Ratio am:pm cortisol 12 months	50	2.09	1.18–3.4	13	1.9	1.33–3.26	0.97

The *p* values of less than 0.05 are marked with an asterisk. Note that there are no *p* values of < 0.01.

* Significant at the 0.05 level.

** Significant at the 0.01 level.

Table 3 shows correlations between cortisol levels at each time point and delirium measures at the same time point. Afternoon cortisol levels significantly correlated with the three delirium rating scores (DRS-R98, DEL-box and CAM-ICU) on day 3 and day 5 after stroke and correlated with one score (DRS-R98) on day 14 and with two (DEL-box and CAM-ICU) on day 28. Morning cortisol levels significantly correlated with the three delirium rating scores on day 14 and with the CAM-ICU alone on day 28. The ratio of morning to afternoon cortisol correlated significantly with the CAM-ICU on day 3, with all three delirium rating scores on day 5 and with the DRS-R98 on day 28. There were no significant correlations between any of the cortisol measurements taken at 4 months and 12 months and measures of delirium.

**Table 3:** Correlations between cortisol levels at each time point and measures of delirium taken at the same time point.

**Cortisol sample (nmol/L)**	**DRS-R98 (Spearman's rho)**	**Del-box (Spearman's rho)**	**CAM-ICU (biserial correlation)**
9 am cortisol day 3	0.226 (*p* = 0.063)	−0.150 (*p* = 0.182)	0.187 (*p* = 0.104)
3.30 pm cortisol day 3	0.322 (*p* = 0.015)^*^	0.412 (*p* = 0.005)^**^	−0.66 (*p* < 0.001)^***^
Ratio am:pm cortisol day 3	−0.182 (*p* = 0.121)	0.167 (*p* = 0.161)	−0.256 (*p* = 0.049)^*^
9 am cortisol day 5	0.07 (*p* = 0.29)	0.021 (*p* = 0.43)	−0.010 (*p* = 0.47)
3.30 pm cortisol day 5	0.30 (*p* = 0.007)^**^	0.259 (*p* = 0.019)^*^	−0.239 (*p* = 0.03)^*^
Ratio am:pm cortisol day 5	0.273 (*p* = 0.01)^**^	0.336 (*p* = 0.004)^**^	0.34 (*p* = 0.003)^**^
9 am cortisol day 7	0.067 (*p* = 0.318)	0.029 (*p* = 0.424)	−0.176 (*p* = 0.106)
3.30 pm cortisol day 7	0.126 (*p* = 0.200)	−0.344 (*p* = 0.012)	−0.006 (*p* = 0.483)
Ratio am:pm cortisol day 7	0.071 (*p* = 0.321)	0.260 (*p* = 0.051)	0.132 (*p* = 0.194)
9 am cortisol day 14	0.343 (*p* = 0.02)^*^	−0.394 (0.012)^*^	0.453 (*p* = 0.003)^**^
3.30 pm cortisol day 14	0.369 (*p* = 0.025)^*^	−0.144 (0.233)	0.131 (0.249)
Ratio am:pm cortisol day 14	0.171 (*p* = 0.201)	−0.315 (*p* = 0.062)	0.195 (*p* = 0.170)
9 am cortisol day 28	−0.135 (*p* = 0.121)	−0.175 (*p* = 0.076)	0.234 (*p* = 0.022)^*^
3.30 pm cortisol day 28	0.167 (*p* = 0.077)	−0.37 (*p* = 0.001)^**^	0.208 (*p* = 0.041)^*^
Ratio am:pm cortisol day28	0.330 (*p* = 0.004)^**^	0.209 (*p* = 0.059)	−0.104 (*p* = 0.210)
9 am cortisol 4 months	0.090 (*p* = 0.243)	0.080 (*p* = 0.267)	0.180 (*p* = 0.155)
3.30 pm cortisol 4 months	0.100 (*p* = 0.204)	0.092 (*p* = 0.220)	NA
Ratio am:pm cortisol 4 months	−0.072 (*p* = 0.299)	0.068 (*p* = 0.308)	NA
9 am cortisol 12 months	0.008 (*p* = 0.008)	−0.016 (*p* = 0.449)	0.050 (*p* = 0.345)
3.30 pm cortisol 12 months	0.205 (*p* = 0.050)	−0.086 (*p* = 0.246)	0.168 (*p* = 0.089)
Ratio am:pm cortisol 12 months	−0.186 (*p* = 0.074)	0.047 (*p* = 0.358)	−0.076 (0.279)

* Significant at the 0.05 level.

** Significant at the 0.01 level.

*** Significant at the < 0.001 level.

NA, not applicable as CAM-ICU negative for all subjects.

The assumptions for logistic regression including linearity and independence of errors were tested and met. Multicollinearity was tested using variance inflation factor statistics. There was no independent association between cortisol and delirium on multivariable analyses (Table 4).

**Table 4:** Summary of the Multivariable analysis showing the independent predictors of delirium.

**Cortisol measure**	**Number of participants**	**Proportion of variance accounted for (%)**	**Statistically significant predictors (*p* < 0.05) of delirium**
Median 9 am cortisol (days 0–7):	86	33–48	Age NIHSS on admission
Median 3.30 pm cortisol (days 0–7):	83	27–40	Age NIHSS on admission
Ratio of median morning to afternoon cortisol (days 0–7):	83	30–45	NIHSS on admission
Peak 9 am cortisol (days 0–14)	84	37 to 55	Age NIHSS on admission IQCODE score
Peak 3.30 pm cortisol (days 0–14)	82	27 to 41	Age
Ratio of peak morning: afternoon cortisol (days 0–14)	80	34 to 52	Age NIHSS on admission IQCODE APACHE II

Table 5 shows the univariate association between cortisol and clinical variables. Note that *p* values are specified as being significant at the 0.05 level, the 0.01 level and the < 0.001 level.

**Table 5:** Correlations between cortisol levels and baseline characteristics.

**Cortisol sample (nmol/L)**	**Age (years)**	**NIHSS score on admission**	**APACHE II score on admission**	**IQCODE score**	**Charlson comorbidities index score**	**Sex**
9 am cortisol day 3	0.357 (*p* = 0.013)^*^	0.425(*p* = 0.003)^**^	0.503 (*p* = < 0.001)^***^	0.041 (*p* = 0.798)	0.143 (*p* = 0.331)	0.187 (*p* = 0.203)
3.30 pm cortisol day 3	0.168 (*p* = 0.269)	0.381 (*p* = 0.010)^*^	0.362 (*p* = 0.015)^*^	−0.108 (*p* = 0.505)	−0.054 (*p* = 0.725)	0.230 (*p* = 0.129)
Ratio am:pm cortisol day 3	−0.002 (*p* = 0.988)	0.009 (*p* = 0.955)	0.159 (*p* = 0.308)	0.004 (*p* = 0.983)	0.159 (*p* = 0.309)	0.203 (*p* = 0.191)
9 am cortisol day 5	0.297 (*p* = 0.012)	0.139 (*p* = 0.251)	0.232 (*p* = 0.054)	−0.066 (*p* = 0.609)	0.012 (*p* = 0.920)	−0.085 (*p* = 0.486)
3.30 pm cortisol day 5	0.392 (*p* = 0.001)^**^	0.075 (*p* = 0.547)	0.237 (*p* = 0.055)	0.085 (*p* = 0.513)	0.02 (*p* = 0.874)	−0.098 (*p* = 0.432)
Ratio am:pm cortisol day 5	−0.087 (*p* = 0.493)	−0.091 (*p* = 0.477)	−0.091 (*p* = 0.472)	−0.017 (*p* = 0.898)	0.046 (*p* = 0.720)	0.062 (*p* = 0.626)
9 am cortisol day 7	−0.043 (*p* = 0.762)	−0.170 (*p* = 0.229)	−0.024 (*p* = 0.868)	0.024 (*p* = 0.878)	−0.174 (*p* = 0.216)	−0.036 (*p* = 0.797)
3.30 pm cortisol day 7	0.139 (*p* = 0.351)	−0.050 (*p* = 0.737)	0.243 (*p* = 0.099)	−0.150 (*p* = 0.357)	−0.104 (*p* = 0.486)	−0.248 (*p* = 0.093)
Ratio am:pm cortisol day 7	−0.144 (*p* = 0.346)	−0.118 (*p* = 0.439)	−0.164 (*p* = 0.281)	0.082 (*p* = 0.623)	−0.197 (*p* = 0.195)	0.149 (*p* = 0.329)
9 am cortisol day 14	0.338 (*p* = 0.041)	−0.052 (*p* = 0.459)	0.303 (*p* = 0.068)	0.134 (*p* = 0.459)	0.072 (*p* = 0.670)	0.338 (*p* = 0.041)^*^
3.30 pm cortisol day 14	0.537 (*p* = 0.002)	0.225 (*p* = 0.232)	0.419 (*p* = 0.021)^*^	0.173 (*p* = 0.379)	0.084 (*p* = 0.658)	0.064 (*p* = 0.737)
Ratio am:pm cortisol day 14	−0.006 (*p* = 0.978)	−0.193 (*p* = 0.336)	−0.012 (*p* = 0.954)	0.147 (*p* = 0.482)	−0.144 (*p* = 0.473)	0.218 (*p* = 0.275)
9 am cortisol day 28	0.049 (*p* = 0.672)	0.046 (*p* = 0.691)	0.038 (*p* = 0.740)	−0.010 (*p* = 0.936)	−0.074 (*p* = 0.521)	−0.001 (*p* = 0.996)
3.30 pm cortisol day 28	0.390 (*p* = 0.001)	0.233 (*p* = 0.045)^*^	0.357 (*p* = 0.002)^**^	−0.048 (*p* = 0.698)	0.273 (*p* = 0.019)	−0.182 (*p* = 0.120)
Ratio am:pm cortisol day28	−0.203 (*p* = 0.105)	−0.213 (*p* = 0.089)	−0.220 (*p* = 0.078)	0.008 (*p* = 0.950)	−0.219 (*p* = 0.08)	−0.11 (*p* = 0.932)
9 am cortisol 4 months	0.168 (*p* = 0.181)	0.122 (*p* = 0.333)	−0.078 (*p* = 0.536)	0.154 (*p* = 0.236)	−0.045 (*p* = 0.721)	−0.123 (*p* = 0.328)
3.30 pm cortisol 4 months	0.072 (*p* = 0.541)	0.109 (*p* = 0.356)	0.113 (*p* = 0.336)	0.130 (*p* = 0.291)	0.307 (*p* = 0.008)^**^	−0.140 (*p* = 0.234)
Ratio am:pm cortisol 4 months	0.033 (*p* = 0.805)	−0.070 (*p* = 0.600)	−0.165 (*p* = 0.212)	−0.011 (*p* = 0.937)	−0.251 (*p* = 0.055)	−0.112 (*p* = 0.400)
9 am cortisol 12 months	−0.250 (*p* = 0.04)	0.065 (*p* = 0.598)	−0.252 (*p* = 0.038)^*^	−0.053 (*p* = 0.675)	0.053 (*p* = 0.669)	−0.094 (*p* = 0.445)
3.30 pm cortisol 12 months	0.094 (*p* = 0.447)	0.068 (*p* = 0.587)	−0.051 (*p* = 0.681)	0.103 (*p* = 0.422)	0.207 (*p* = 0.093)	−0.105 (*p* = 0.399)
Ratio am:pm cortisol 12 months	−0.169 (*p* = 0.185)	−0.012 (*p* = 0.925)	−0.058 (*p* = 0.651)	−0.138 (*p* = 0.297)	−0.129 (*p* = 0.313)	−0.308 (*p* = 0.014)^*^

* Significant at the 0.05 level.

** Significant at the 0.01 level.

*** Significant at the < 0.001 level.

## Discussion

This study is the largest, and most detailed, study to date, to examine the associations between cortisol levels and delirium in the first 12 months after stroke. In bivariate analyses, higher afternoon cortisol levels, but not morning cortisol, were associated with delirium and its severity in the first 14 days after stroke. However, our multivariable analyses showed that only age and NIHSS score were independently associated with delirium. This is a challenging area of research and thus our study contributes valuable data to the field.

It is possible that we missed a small effect size because we did not recruit our target of 120 patients and the incidence of delirium was lower than predicted, possibly because of improvements in stroke unit care that had occurred by the time of the study. Nevertheless, the incidence of delirium was still in the range of previous studies of delirium frequency. Also it is possible that we might have missed short episodes of delirium between our assessments, particularly if the delirium was hypoactive, but we did use the Chart CAM, which includes questions about fluctuation and changes in cognition on the non-assessed days. We did not have baseline cortisol levels pre-stroke-and therefore could not report changes from baseline to after the acute stroke. It is possible that changes in cortisol levels from baseline-rather than the absolute values after stroke-might have been relevant to the etiology of delirium. Although we found no association between delirium and cortisol dysregulation in the entire group, it is possible that there were subgroups with specific constellation of demographics and stroke characteristics where cortisol dysregulation is relevant.

A larger study with greater power would be needed to establish whether cortisol truly has an independent effect on delirium status, or whether it is simply a marker of other factors, such as stroke severity. It must be noted that large studies in this patient population are difficult to do, because participants are often very unwell following stroke and yet must be assessed for delirium and have cortisol levels measured regularly. Proxy consent is often required (either because of the severity of stroke or because of pre-existing dementia) and this, along with the fact that a long-term follow-up period is desirable (because delirium can persist) make recruitment challenging. These difficulties are reflected in the small sample sizes in previous studies. Meta-analysis would also be challenging with the studies currently available, as methodologies employed vary considerably from study to study, particularly in the method of cortisol measurement. We chose to assess cognition at 4 months after stroke, and then again at 12 months; whilst additional measurements at, say every 3 months, would have made our data easier to combine in a meta-analysis, more frequent measures would not have been practical for us to perform, and could have led to greater attrition before 12 months. Also, previous studies in this field had assessed patients at 4 months.

Stroke severity (as measured by the NIHSS score) and age were both independently associated with delirium in this study. This is interesting, and supports the existing evidence with respect to predictors of delirium after stroke. A large study of delirium after stroke (*n* = 527) found that stroke severity and age were independent predictors of delirium (although age only became an independent predictor if brain atrophy was left out of the model, presumably because the two may co-vary), and as previously described these factors both have plausible pathophysiological mechanisms which might explain their consistent association with delirium (Oldenbeuving et al., 2011). Age is associated with neurone loss, reduced blood flow to the brain, and reduced vascular density in the brain, all of which may increase susceptibility to delirium. Pathological processes in the brain such as those seen in vascular dementia or Alzheimer's dementia are also more common in the aging brain, and these also increase the risk of delirium (Maldonado, 2013). Stroke severity is a complex variable, as the severity score may not always correlate with the actual size of the brain lesion, although in many cases it will. Furthermore, stroke severity is not necessarily a marker of overall illness severity, although again there is often a correlation between the two (and those with a large TACS are also more at risk of intercurrent illness, such as pneumonia). However, the stroke severity score is a marker of the direct brain insult and subsequent brain tissue damage and loss (Mitsias et al., 2002), all of which are likely to increase the risk of delirium.

Ahmed and colleagues (Ahmed et al., 2004) examined salivary cortisol levels after stroke (rather than plasma or urinary levels). They found a mean salivary cortisol level (at admission) of 18.4 nmol/L in the morning and 6.7 nmol/L in the afternoon. In the current study, we found a mean morning level of 25.9nmol/L and a mean afternoon level of 17.7 nmol/L during the first week after stroke. The reason for the slightly higher levels in this study are not clear. It may partly be accounted for by the fact that collection devices and assays used differed, however assays in this study were calibrated to industry standards. Stroke severity is known to influence cortisol levels in a bidirectional fashion, in that those with very severe strokes in ICU have been shown to have low cortisol levels and lose the diurnal circadian rhythm, whereas more generally the more severe the stroke, the higher the cortisol level is (Barugh et al., 2014). The mean NIHSS score in the Ahmed study was 7, and the median NIHSS in this study was 5, indicating similar stroke severity. The Ahmed study (Ahmed et al., 2004) was smaller (*n* = 58) and the study participants were younger (mean age of 66, compared with 77 in this study), and although delirium wasn't reported, its incidence is likely to have been lower, because participants were younger (and therefore less likely to have underlying risk factors for delirium such as pre-existing dementia).

There are also a few relevant studies about cortisol dysregulation and its association with delirium in conditions other than stroke. Witlox et al. performed cerebrospinal fluid (CSF) cortisol measurements in 75 patients of patients about to undergo surgical repair of acute hip fracture, and found no association between cortisol levels and subsequent delirium (Witlox et al., 2020), whilst Hall et al. found that in 148 patients with hip fracture, CSF cortisol was higher in those with prevalent delirium (Hall et al., 2015). A 2023 systematic review of 315 studies recruiting 101 144 patients (Ormseth et al., 2023) which reported the predisposing and precipitating factors for delirium did not include cortisol dysregulation in the list of relevant factors-though this review did not include the Witlox (Witlox et al., 2020) and Hall (Hall et al., 2015) studies. A 2023 study on the intensive care unit in 76 patients found that those who developed delirium had lost the normal circadian rhythm in cortisol and melatonin (Li et al., 2023). It is not clear whether, and if so how, the results of our study might be applicable to conditions other than stroke.

There are some limitations to our study. Our sample size was small and we did not reach our target of 120 patients. Participants were recruited as soon after stroke as possible, however because the time of stroke onset was taken to be when the participant was last seen well, there were delays between this time and first assessment. There were also delays when proxy consent was required, as proxies, understandably, often required 24 h or more to consider the study before granting consent. These factors meant that some participants were not assessed, nor did they have salivary cortisol measured until day 5 after stroke onset. Participants were not assessed every day following recruitment, rather they were assessed on alternate days. This methodology was used to reduce the burden of assessments for participants-and thus to minimize the number who dropped out. The chart-CAM and clinician and informant information was used to provide information about the days when formal assessments weren't scheduled. It is possible that brief episodes of delirium were missed, although this could also have been the case if participants had been assessed daily. There was selection bias in the study, as proxies were more likely to decline consent compared with potential participants themselves, resulting in a bias toward selection of those with capacity at recruitment. We used the OCSP classification as this had previously been shown to relate to prognosis, lesion location and likely etiology, but we acknowledge that an additional classification system based on vessel occlusion and territory might have been informative too. We used salivary cortisol measurements-salivary cortisol measures free cortisol which is biologically active and is therefore a better “window into the brain” than total circulating cortisol from blood or urine measurements (Clow and Smyth, 2020). However, in a study in 157 healthy volunteers in 2024, there was only a weak correlation (*r* = 0.28) between salivary cortisol and CSF cortisol, and moderate correlation with plasma cortisol (*r* = 0.49) (Katharine et al., 2025). In our study, it would have been impractical to perform lumbar punctures to obtain CSF, but in the future, researchers may wish to consider plasma sampling twice a day (though this would require venopuncture rather than saliva collection). Finally, as discussed above, the sample size was small, although this remains the largest study to date of cortisol and delirium after stroke.

In summary, we did not find evidence of an independent association between salivary cortisol levels after stroke and the development of delirium. Because the study was slightly underpowered, we cannot exclude for certain a relationship, but if such a relationship does exist, then it is likely to be small, and may not be of clinical relevance. Our data could be used to inform the design of future larger definitive studies. The focus of future studies should probably be on alternative mechanisms for delirium after stroke. There are multiple plausible triggers that have been described in the general population (Ormseth et al., 2023). After stroke, the interplay between neurotransmitters such as dopamine, acetylcholine, serotonin, glutamate, and gamma-Aminobutyric acid, and neuroinflammation including interleukins and interferons is worthy of study (Kneihsl et al., 2024).

## Data Availability

The datasets presented in this article are not readily available because data have been archived. Requests to access the datasets should be directed to gillian.e.mead@ed.ac.uk.
